# Outcomes in patients with tether rupture after anterior vertebral tethering for adolescent idiopathic scoliosis: the good, the bad, and the ugly

**DOI:** 10.1007/s43390-025-01077-0

**Published:** 2025-03-28

**Authors:** John T. Braun, Sofia C. Federico, David M. Lawlor, Brian E. Grottkau

**Affiliations:** 1 Massachusetts General Hospital, 55 Fruit St., Yawkey 3E, Boston, MA 02114 USA; 2 Massachusetts General Hospital, Harvard Medical School, Boston, MA USA

**Keywords:** Anterior vertebral tethering, Adolescent idiopathic scoliosis, Tether rupture, Complications, Outcomes

## Abstract

**Introduction:**

Though multiple studies have reported tether rupture rates after anterior vertebral tethering (AVT) as high as 50%, few have adequately analyzed the clinical significance of tether rupture and factors that potentially increase the likelihood of revision surgery. We reviewed 262 consecutive adolescent idiopathic scoliosis (AIS) patients after AVT with the goal of identifying early and late tether ruptures and categorizing these tether ruptures as inconsequential, consequential, problematic, or beneficial. Our hypothesis was that the tether rupture rate after AVT for AIS would be significant but only a small percentage of patients would require revision surgery.

**Methods:**

Charts, radiographs, and CT scans were reviewed for tether rupture in 262 consecutive AIS patients treated with AVT for thoracic and thoracolumbar/lumbar curves 33–77°. Early tether rupture occurred < 2 years and late tether rupture ≥ 2 years postoperatively. Tether rupture was further categorized as inconsequential (final curve < 40° and no pain), consequential (curve ≥ 40° and/or convex back pain), problematic (revision surgery required), or beneficial (improvement of overcorrection) at follow-up.

**Results:**

Of 262 consecutive AIS patients status post AVT (106 thoracic curves, 53 thoracolumbar curves, and 103 double curves), tether rupture was found in 45 patients with 66 curves (34 thoracic and 32 thoracolumbar/lumbar) treated at age 14.5 years and at Risser 2.6 and Sanders 4.7. Curves with tether rupture corrected from 50.3° preoperatively to 20.8° postoperatively, but lost 7.2° of correction with tether rupture settling at 28.0° final at 2.6 years (0–11 years). Early tether rupture occurred in 12/133 (9%) and late tether rupture in 33/129 (26%) patients with 2–11 year follow-up. Tether rupture was inconsequential in 67% (30/45) of patients, consequential in 13% (6/45), problematic in 16% (7/45), and beneficial in 4% (2/45). In those patients with tether rupture, 69% occurred in a thoracolumbar/lumbar curve and 47% demonstrated a rupture at L2,3. Revision surgery for a thoracolumbar/lumbar tether rupture involved tether replacement alone in 4 patients and thoracic fusion in 2 additional patients, 1 requiring thoracic fusion alone, and 1 requiring thoracic fusion with thoracolumbar/lumbar tether replacement (hybrid). Revision surgery for a thoracic tether rupture involved 1 tether replacement and 1 thoracic fusion. Revision surgery was unrelated to curve correction or loss of correction, but was related to multiple tether ruptures and convex back pain (*p* < 0.005).

**Conclusion:**

This study demonstrated an early tether rupture rate of 9% and late tether rupture rate of 26% in a large series of patients treated with AVT for AIS over 14 years. While the majority of patients had inconsequential tether rupture (67%), with 7.2° loss of correction, a final curve < 40°, and no pain, a number of patients had consequential (13%) or problematic tether rupture (16%). These adversely affected patients had a final curve ≥ 40°, convex back pain, or required revision surgery. Additionally, a small number of patients (4%) actually benefitted from tether rupture by improvement in an area of impending overcorrection.

**Level of evidence:**

IV.

## Introduction

Anterior vertebral tethering (AVT) has been proposed as a minimally invasive alternative to fusion surgery for adolescent idiopathic scoliosis (AIS) as it offers the potential for definitive scoliosis treatment with the possibility of preservation of growth, motion, function, and overall health of the spine. Though outcomes after AVT for AIS have improved over the past decade with better indications and techniques, tether rupture has remained a persistent problem. With tether rupture rates reported in the 50% range or greater, the threat to long-term correction and control of scoliosis is significant [1–6]. However, despite the high rate of tether rupture, many patients tolerate tether rupture quite well without substantial loss of correction or relentless progression of deformity requiring revision surgery.

The purpose of this study was to evaluate radiographic outcomes after tether rupture in a consecutive series of AIS patients treated with AVT over a 14 year period. Our primary hypothesis was that tether rupture would not be uncommon after AVT but would be well tolerated by most patients. Our secondary hypotheses were that there would be differences in early versus late tether rupture rates and that certain factors would make revision surgery more likely.

## Methods

Under IRB approved protocols, a retrospective analysis was performed on all AIS patients consecutively treated with AVT from 2010 to 2024. The overall range of curve magnitude was 33–77° with skeletal maturity spanning Risser -1 to 5 (Risser -1 indicating Risser 0 with open triradiate cartilages) and Sanders 2–8. Both single- and double-curve patterns were treated in the thoracic and thoracolumbar/lumbar spine spanning Lenke types 1, 2, 3, 5, and 6. Charts and radiographs were reviewed to establish basic demographic data and identify tether ruptures and revision surgeries. All patients had standard preoperative posteroanterior and lateral full length scoliosis radiographs. The Cobb method was used to measure curve magnitude on preoperative, postoperative and final radiographs. Skeletal maturity was assessed using the Risser sign with an additional hand film to assess bone age as warranted.

Patients with suspected tether rupture demonstrated an increased interscrew angle (ISA) of ≥ 5° between 2 screws across a single disc level [4]. Patients with confirmed tether rupture demonstrated a visible disruption in tether integrity on CT imaging and/or on direct inspection during revision surgery. Suspected and confirmed tether ruptures were considered inconsequential at follow-up in patients with a final curve magnitude of < 40° and no pain. Tether ruptures were considered consequential in patients with a final curve ≥ 40° and/or convex back pain and were considered problematic in patients in whom revision surgery was required. Tether ruptures were considered beneficial in those patients who demonstrated improvement in an area of impending overcorrection.

The surgical technique for this procedure has been described previously by our group [7–9]. All patients were placed in a lateral decubitus position for surgery. A 3-portal endoscopic approach with single lung ventilation was used for thoracic curves and a mini-open thoracoabdominal approach for thoracolumbar/lumbar curves. The diaphragm was preserved in all cases. Segmental vessels were sacrificed prior to bicortical screw placement under fluoroscopic guidance. During the first 12 years of this study, all levels were instrumented with a single PET cord spanning at least Cobb end vertebra to end vertebra. During the last 2 years of this study, a double PET cord was used in most thoracolumbar/lumbar curves. The PET cord was tensioned under fluoroscopic guidance to achieve the desired correction of disc angulation at each level with the typical goal of achieving a neutral disc. Tensioning proceeded from proximal to distal with maximal tension applied to the majority of the curve, including the apex, with less tension at the ends. A temporary chest tube was used to assist with reinflation of the lung and evacuation of blood and fluid from the chest but was usually removed, under appropriate circumstances, at the end of the procedure. Spinal cord monitoring was used in all cases. Patients with double-curve patterns had both curves treated under one anesthetic with the thoracic curve treated first. An access surgeon was utilized for all procedures.

Hydroxyapatite-coated titanium screws and a polyethylene-terephthalate (PET) cord from the Dynesys Dynamic Stabilization System (Zimmer Biomet Spine, Broomfield, CO) were used in all cases without the polycarbonate-urethane spacer during the first 10 years of the study. As this device system was approved by the FDA in 2003 for adult lumbar spine stabilization as an adjunct to fusion, its use in the treatment of scoliosis in children during this portion of the study time period was considered an off-label indication. During the last 4 years of the study, hydroxyapatite coated titanium screws and a polyethylene-terephthalate (PET) cord from The Tether System (Highridge, Westminster, CO) were used. This device system achieved FDA approval in 2019.

Standard statistical analysis was performed using paired and one-sided t tests, where appropriate, to determine the significance of preoperative versus postoperative or final radiographic measurements. Chi squared analysis was used to determine the significance of various factors associated with revision surgery. All statistical analyses were conducted in Microsoft Excel for Mac (version 16.52; Microsoft) with an alpha set at 0.05.

## Results

Of 262 consecutive AIS patients treated with AVT, tether rupture was found in 45 patients with 66 curves (34 thoracic and 32 thoracolumbar/lumbar) treated at age 14.5 years with skeletal maturity graded at Risser 2.6 and Sanders 4.7. Patients with tether rupture had curves that initially corrected from 50.3° preoperatively to 20.8° postoperatively, but lost 7.2° (5–40°) of correction on average after tether rupture, settling at 28.0° final at 2.6 years (0–11 years). In the majority of patients (34/45), tether ruptures were suspected after an ISA change of ≥ 5° was demonstrated on a standard posteroanterior radiograph. In the remaining patients (11/45), suspected tether ruptures were confirmed by CT scan and/or direct inspection during revision surgery. Early tether rupture was seen in 12/133 patients (9%) with < 2 year follow-up. These early tether ruptures occurred at 1.0 year on average (0–1.5 years) with a single patient experiencing an immediate tether rupture on POD #2. Late tether ruptures occurred at 3.2 years on average in 33/129 patients (26%) with ≥ 2 year follow-up (2–11 years).

**Fig. 1:**
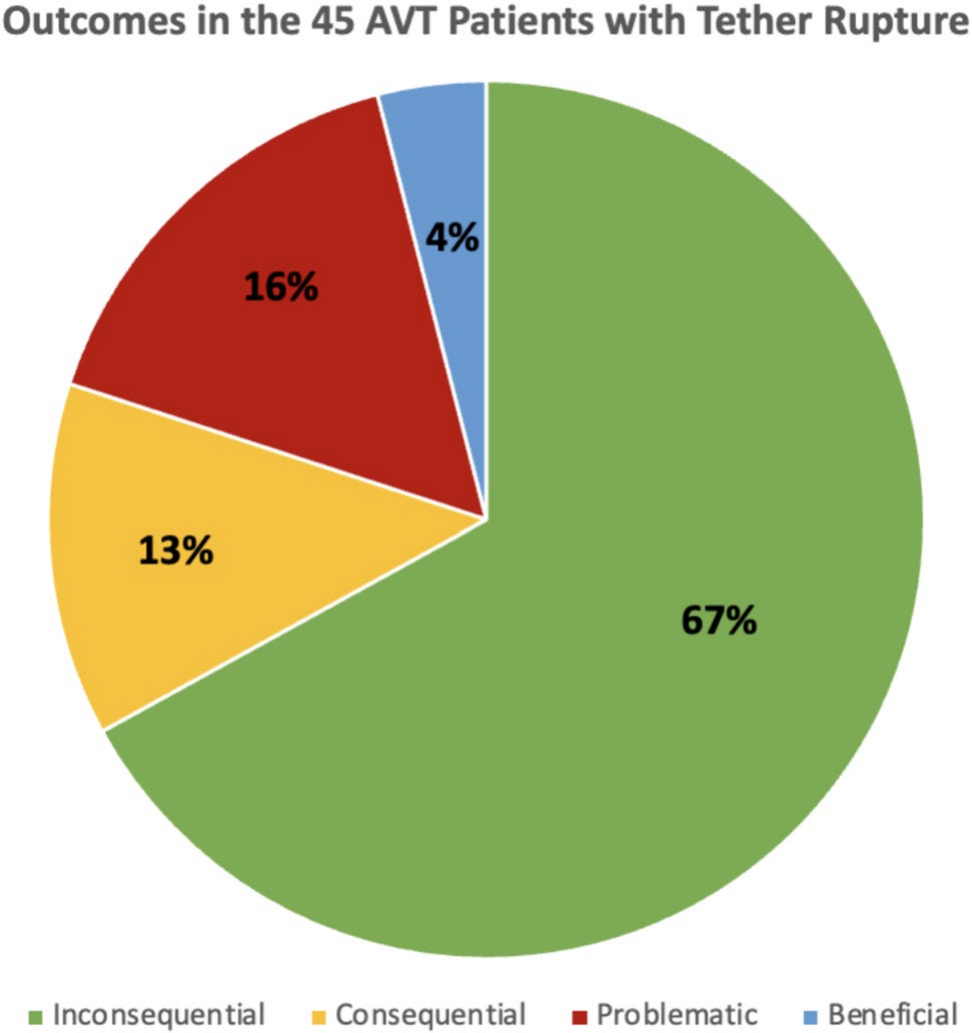
Outcomes in the 45 AVT patients with tether rupture revealed 67% were inconsequential, 13% consequential, 16% problematic, and 4% beneficial

**Fig. 2:**
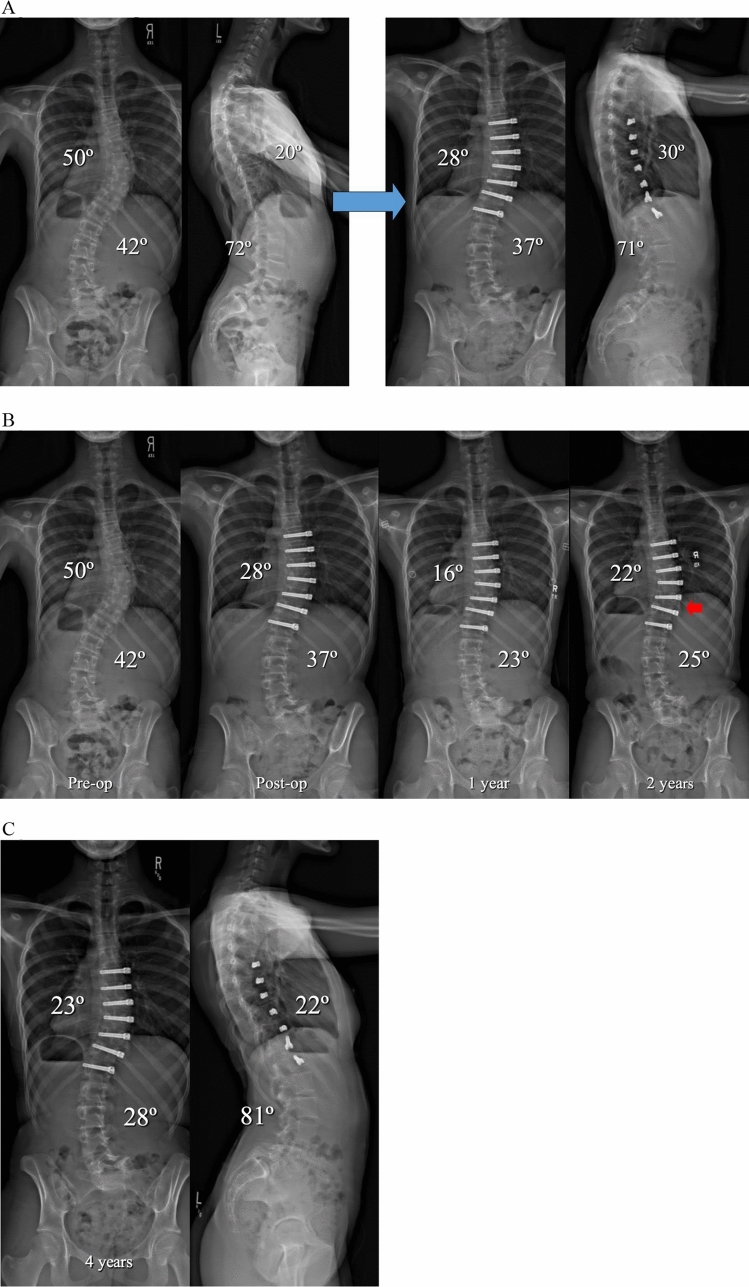
**A** Preoperative and postoperative posteroanterior and lateral radiographs of a 13 + 6-year-old premenarchal female with a Lenke 1B curve pattern involving a 50º curve right T6-T12 and a 42º curve left T12-L4 at Risser 0 and Sanders 5. Initial curve correction after AVT T6-T12 resulted in thoracic curve correction from 50º to 28º and indirect curve correction below from 42º to 37º. A stable and asymptomatic grade 3 spondylolisthesis at L5 was present on the lateral films as well as slight inharmonious sagittal alignment of the distal screws T10-T12. **B** Preoperative, postoperative, 1-year, and 2-year posteroanterior radiographs demonstrated initial curve correction to 28º, subsequent growth modulation to 16º over the first year, and then 6º loss of correction to 23º due to a late inconsequential tether rupture at T10,11 (ISA change 10º) at 2 years (red arrow). After the initial indirect correction of the lumbar curve to 37º, additional improvement to 23º occurred over the first year and was maintained at 2 years at 25º. **C** Posteroanterior and lateral radiographs at 4 years demonstrated well-maintained correction of the thoracic curve at 23º with a slight increase in the thoracolumbar/lumbar curve to 28º

**Fig. 3:**
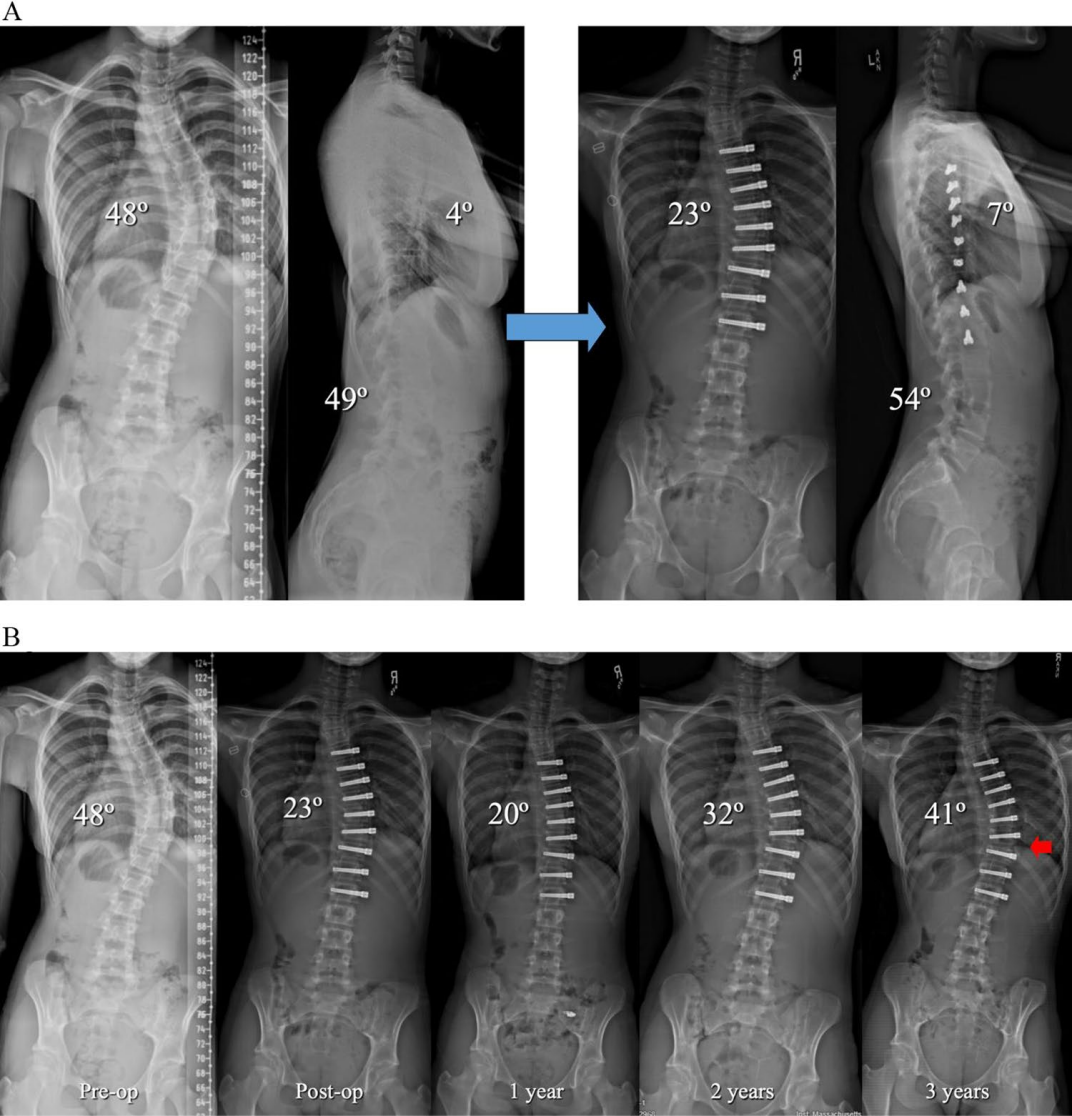
**A** Preoperative and postoperative posteroanterior and lateral radiographs of a 13 + 1 year-old premenarchal female with a Lenke 1AR curve pattern involving a 48º curve right T5-L1 at Risser 1 and Sanders 4. AVT T5-L1 resulted in initial curve correction from 48º to 23º with little change in the thoracic hypokyphosis. **B** Preoperative, postoperative, 1-year, 2-year, and 3-year posteroanterior radiographs demonstrated initial curve correction from 48º to 23º with subsequent improvement to 20º with growth modulation in the first year. A 12º loss of correction was evident at 2 years with another 9º loss of correction at 3 years. Though the 9º angle change at 3 years was correlated with an ISA change of 5º at T10,11, resulting in a consequential tether rupture (red arrow), the prior 12º loss of correction at 2 years did not correlate with an ISA change of ≥ 5º at any single level. This three sport athlete remains active without symptoms and without any interest in revision surgery at this time

**Fig. 4:**
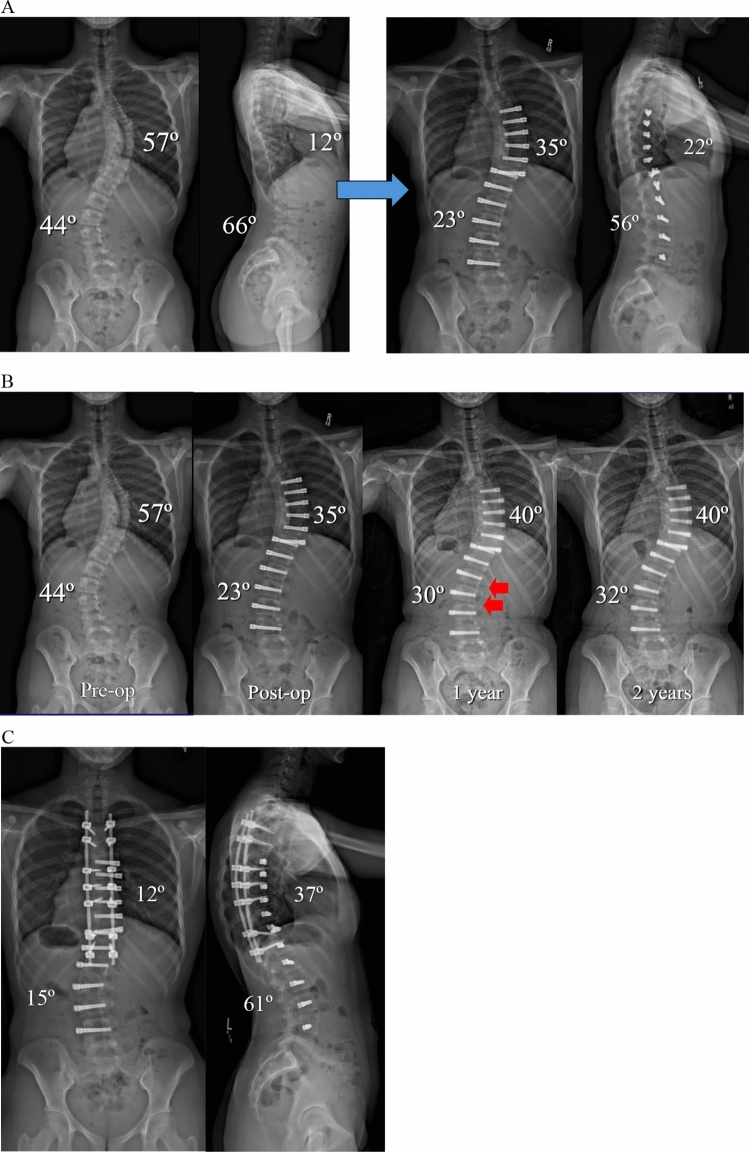
**A** Preoperative and postoperative posteroanterior and lateral radiographs of a 13 + 7 year-old female with a Lenke 1C curve pattern involving a 57º curve right T6-T11 and a 44º curve left T11-L4 at Risser 2 and Sanders 5. AVT T6-T11 and T11-L4 resulted in initial curve corrections from 57º to 35º and 44º to 23º, respectively. **B** Preoperative, postoperative, 1-year, and 2-year posteroanterior radiographs demonstrated initial curve corrections from 57º to 35º and 44º to 23º, respectively, but then loss of 5º and 7º of correction in both curves due to CT confirmed early tether ruptures at L1,2 and L2,3 (ISA changes of 6º at both levels) at 1 year (red arrows) with an additional increase in the thoracic curve to 40º without tether rupture. Though significant additional loss of correction was not demonstrated at 2 years, convex back pain over both curves eventually led to a revision surgery involving selective fusion of the thoracic curve. Thus, these tether ruptures were considered problematic. **C** Preoperative and postoperative posteroanterior and lateral radiographs 3 months status post revision surgery involving posterior spinal fusion T4-T12 without the need for revision of the thoracolumbar/lumbar tether. The resultant hybrid construct was achieved via direct thoracic curve correction from 40º to 12º with indirect curve correction distally from 32º to 15º

**Fig. 5:**
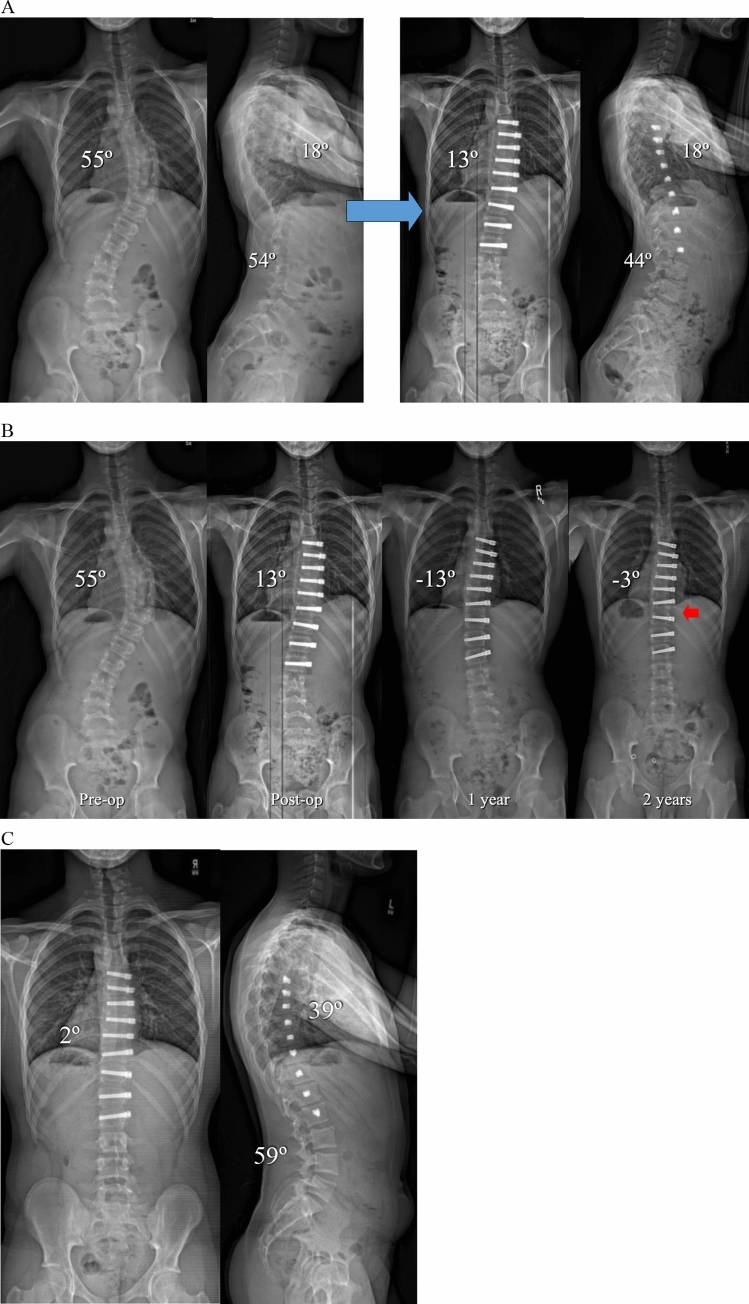
**A** Preoperative and postoperative posteroanterior and lateral radiographs in a 13 + 0-year-old male with a Lenke 1AR curve pattern involving a 55º curve right T6-L2 at Risser -1 and Sanders 2. The initial postoperative correction to 13º was acceptable but slightly beyond the 20º target thought to be optimal to prevent overcorrection in this immature patient. **B** Significant growth modulation over the first year resulted in overcorrection to − 13º that progressed to − 18º at 6 months (poor outside film not shown) but then corrected to − 3º at 2 years after a beneficial tether rupture at T11,12 (red arrow). **C** Posteroanterior and lateral radiographs at 3 years demonstrated well-maintained correction of the thoracic curve at 2 degrees despite the tether rupture at T11,12

Overall, tether rupture was considered inconsequential at follow-up in 67% of patients, consequential in 13%, problematic in 16%, and beneficial in 4% (Fig. 1). In the 67% (30/45) of patients with an inconsequential tether rupture, the final curve magnitude was < 40° (average 25.4°) with no report of associated back pain (Fig. 2). Tether rupture was considered consequential in 13% (6/45) of patients with a final curve magnitude of ≥ 40° (average 44.0°) and/or a report of convex back pain (Fig. 3). Only 1 patient with a consequential tether rupture had a curve < 40° and reported convex back pain. The 16% (7/45) of tether rupture patients considered problematic required revision surgery at an average curve magnitude of 39.8° but with 86% (6/7) reporting convex back pain (Fig. 4). The 4% (2/45) of patients considered to have benefitted from tether rupture had improvement in curve magnitude from -13° to 6° on average in an area of impending overcorrection (Fig. 5).

**Fig. 6:**
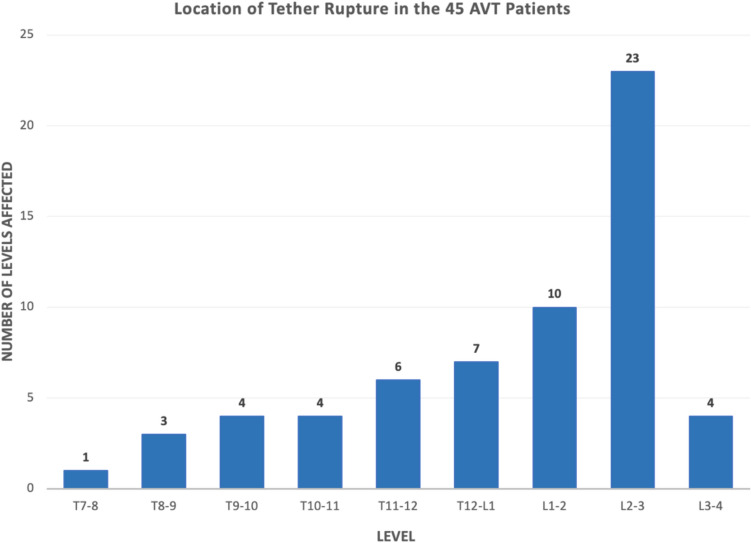
Stratification of tether rupture by location in the 45 AVT patients with tether rupture revealed the majority of tether ruptures occurred in the thoracolumbar/lumbar region (44/62 or 71%). L2,3 was the most commonly affected level (23/62 or 37% of tether ruptures and 21/45 or 47% of patients)

In the 45 AIS patients with tether rupture after AVT, 69% (31/45) were found in thoracolumbar/lumbar curves. Of 63 levels affected, spanning T7,8 to L3,4, the L2,3 level was the most commonly affected level in 47% of patients (Fig. 6). Tether rupture was not only more common in patients with thoracolumbar/lumbar curves (31/45 or 69%) and at L2,3, but 64% of tether rupture patients with a single thoracolumbar/lumbar curve pattern demonstrated rupture at L2,3 and 81% of tether rupture patients with a double-curve pattern demonstrated a rupture at L2,3. While 65% (29/45) of patients demonstrated tether rupture at only 1 level, 31% (14/45) had tether ruptures at 2 levels, and 4% (2/45) had tether ruptures at 3 levels.

**Fig. 7:**
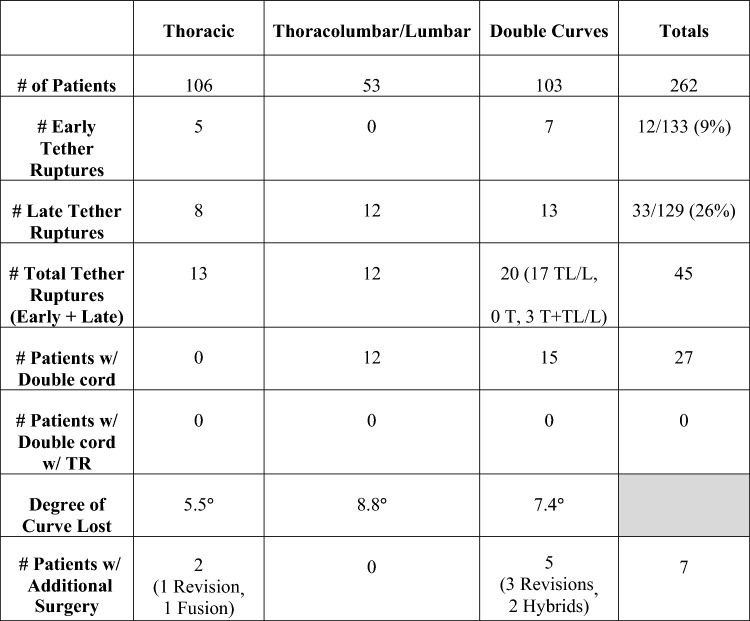
The impact of tether rupture on curve magnitude and the requirement for revision surgery is provided for the three main curve patterns treated in this study. Tether ruptures were more common in patients with single thoracolumbar/lumbar curve or double-curve patterns than in patients with a single thoracic curve pattern. Revision surgeries were more common in double curves than thoracic curves but absent in patients with single thoracolumbar/lumbar curve patterns

Overall, seven additional procedures were performed for tether rupture: 4 tether revisions alone, 2 thoracic fusions, and 1 hybrid procedure (thoracic fusion with thoracolumbar/lumbar tether revision) (Fig. 7). In the 5 patients with tether rupture in a thoracolumbar/lumbar curve, 3 revisions were performed with replacement of the tether using a single cord in 2 patients and a double cord in 1 patient. Two additional revisions were performed for tether rupture in a thoracolumbar/lumbar curve in patients with Lenke 1C double-curve patterns. One of these patients required thoracic fusion alone without revision of the thoracolumbar/lumbar tether, while the other required a hybrid procedure involving thoracic fusion with revision of the thoracolumbar/lumbar tether using a double cord. In the 2 patients with tether rupture in a thoracic curve, 1 revision was accomplished using a double cord, while the other required conversion to fusion due to a combination of large curve magnitude and stiffness as well as significant rib prominence with associated pain. Revision surgery was not found to be related to the amount of initial curve correction or the loss of correction after tether rupture but was found to be related to convex back pain in the region of the rupture. Convex back pain was present in 86% (6/7) of patients requiring revision surgery but only present in 8% (3/38) of the remaining tether rupture patients (*p* < 0.05). Additionally, patients with multiple tether ruptures had an increased incidence of associated convex back pain and, consequentially, the requirement for revision surgery (*p* < 0.005).

## Discussion

**Fig. 8:**
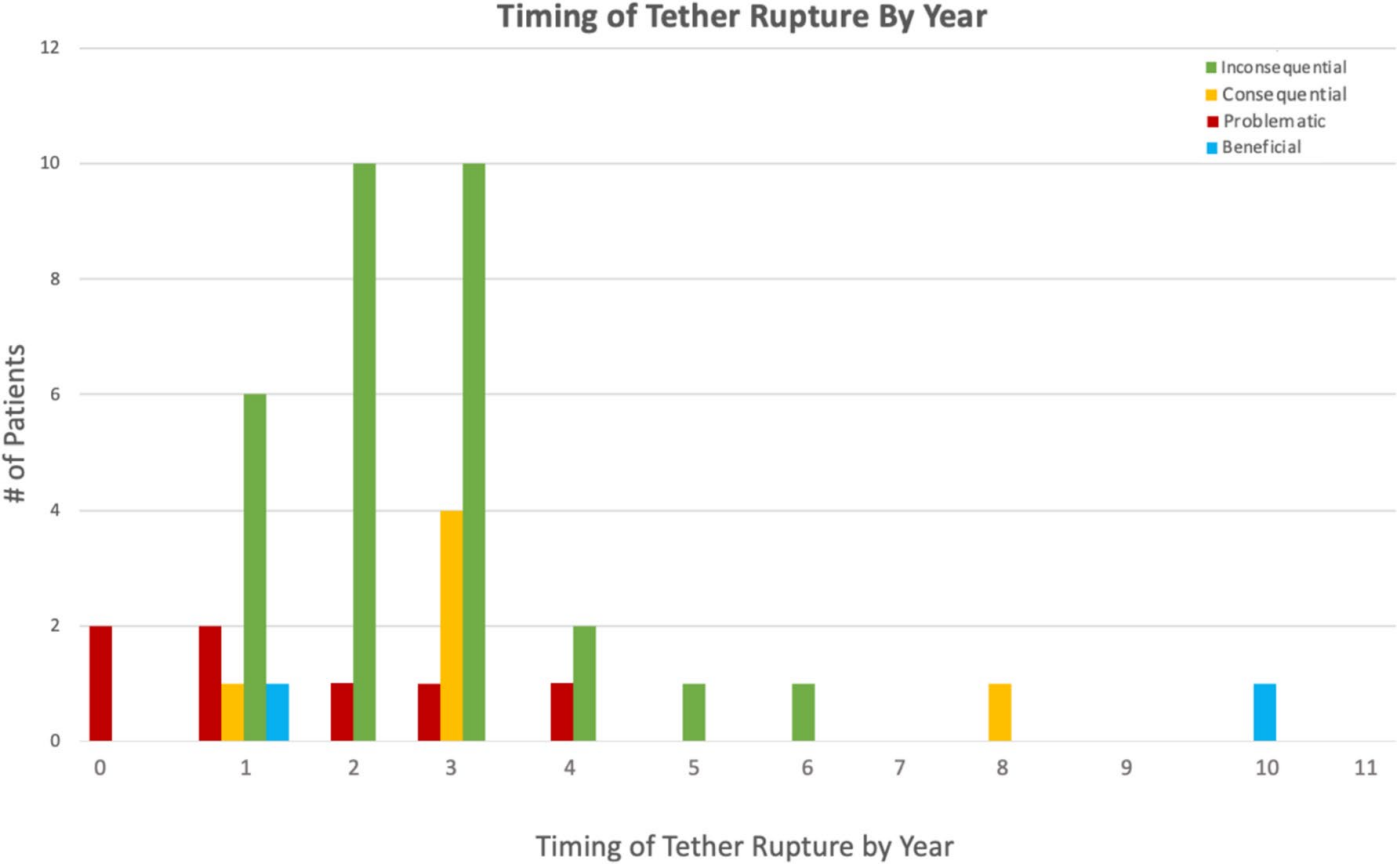
The number of tether ruptures and the specific type of tether rupture are provided for each year over 10 years for all 45 patients with tether rupture. Early tether rupture was demonstrated in 12 of 133 patients (9%) with < 2-year follow-up and in 33 of 129 patients (26%) with ≥ 2 year follow-up. A peak in the overall number of tether ruptures is demonstrated at 2 and 3 years coincident with a peak in inconsequential tether ruptures during that same time. Problematic tether ruptures peaked in the first 2 years but tapered over the next several years. Though no problematic tether ruptures were seen after the 4-year timepoint, this could be related, in part, to a reduction in the number of patients available with long-term follow-up beyond 4 years. For this reason, some additional information on the number of patients with follow-up by year is warranted: 17 patients had < 1-year follow-up, 116 had < 2 years, 66 had 2 years, 32 had 3 years, 12 had 4 years, 7 had 5 years, 4 had 6 years, 4 had 7 years, and 4 had 8–11 years

**Fig. 9:**
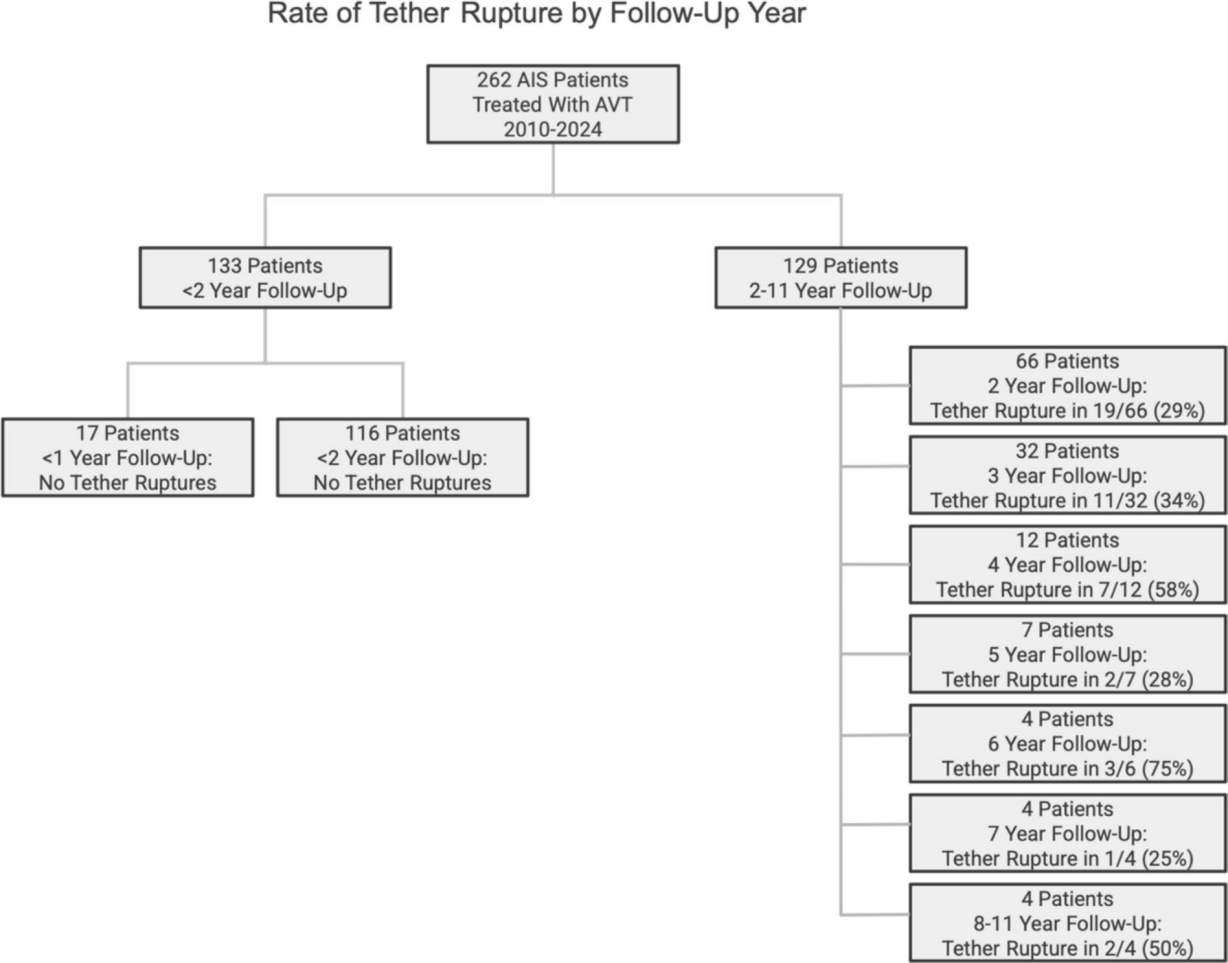
Rate of tether rupture by follow-up year

In this study of 262 consecutive AIS patients treated with AVT over 14 years, 45 patients demonstrated at least 1 tether rupture with 16 of these patients experiencing multiple tether ruptures. Though the early tether rupture rate in this study was only 9% in the first 2 years, this increased to 26% in patients with 2–11 year follow-up, for an overall tether rupture rate of 35%. Given the significant increase in the tether rupture rate after the 2 year timepoint, it is possible that the tether rupture rate will increase further with longer follow-up in a larger cohort of patients (Figs. 8 and 9). However, despite the significant overall rate of tether rupture in these patients, 71% were not adversely affected by their tether rupture during the study period as this occurrence was inconsequential in 67% and, actually, beneficial in 4%. Patients with an inconsequential tether rupture had final curves that averaged 25.4° (range 12–36°) with no report of convex back pain. And patients with a beneficial tether rupture demonstrated an improvement in curve magnitude from -13° to 6° on average in an area of impending overcorrection.

Unfortunately, 29% of the tether rupture patients in this study were adversely affected by their tether rupture as this occurrence was consequential in 13% and problematic, requiring revision surgery, in 16%. In those patients with a consequential tether rupture, resulting in a curve ≥ 40° and/or convex back pain, the average final curve magnitude was 46.7° (range 30–55°) with 33% (2/6) of these patients reporting convex back pain. Though none these patients have reached the point of contemplating revision surgery, it remains a risk. In those patients with a problematic tether rupture, requiring additional surgery, the average final curve magnitude was 39.8° (range 28–57°) with 86% (6/7) of these patients also reporting convex back pain in the region of the tether rupture. The majority of patients requiring additional surgery were treated with tether revision alone (4/7), with the remainder (3/7) requiring conversion to fusion (2 thoracic fusions, 1 hybrid). Though all three fusions were performed in the thoracic region, only one was performed for a single thoracic curve pattern (Lenke 1A). The other 2 thoracic fusions were performed in patients with a double-curve pattern (Lenke 1C) that, despite tether rupture in the thoracolumbar/lumbar curve, demonstrated loss of correction in both curves. One of these patients required thoracic fusion alone without revision of the ruptured thoracolumbar/lumbar tether, while the other required a hybrid procedure involving thoracic fusion with revision of the thoracolumbar/lumbar tether using a double cord.

**Fig. 10:**
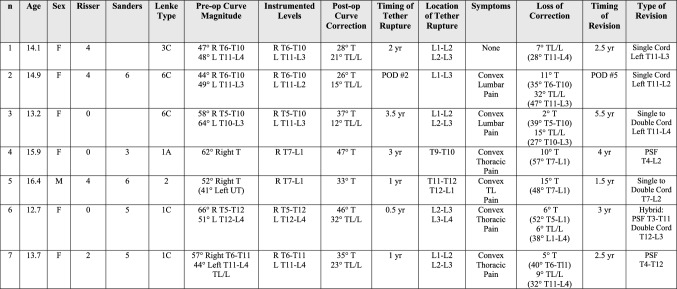
Demographic and radiographic data for the seven patients with problematic tether rupture requiring revision surgery

Of multiple factors potentially thought to be associated with the occurrence of tether rupture, only treatment of a thoracolumbar/lumbar curve demonstrated a trend toward significance with 69% of tether ruptures occurring in these curves. Though previous reports [1, 6] have noted a similar increased incidence of tether rupture in thoracolumbar/lumbar curves, the exact reason for this is not entirely clear but may be related to increased stress on the tether from greater loads in the lower torso and greater motion in a flexible lumbar spine. Of multiple factors potentially associated with revision surgery after tether rupture, two factors reached significance in this study, multiple tether ruptures and convex back pain. Multiple tether ruptures and convex back pain were significantly associated with the requirement for revision surgery (*p* < 0.005). Revision surgery was not found in this study to be associated with curve magnitude, curve location, number of curves treated, initial scoliosis correction, subsequent loss of scoliosis correction after tether rupture, or early versus late tether rupture (Fig. 10).

**Fig. 11:**
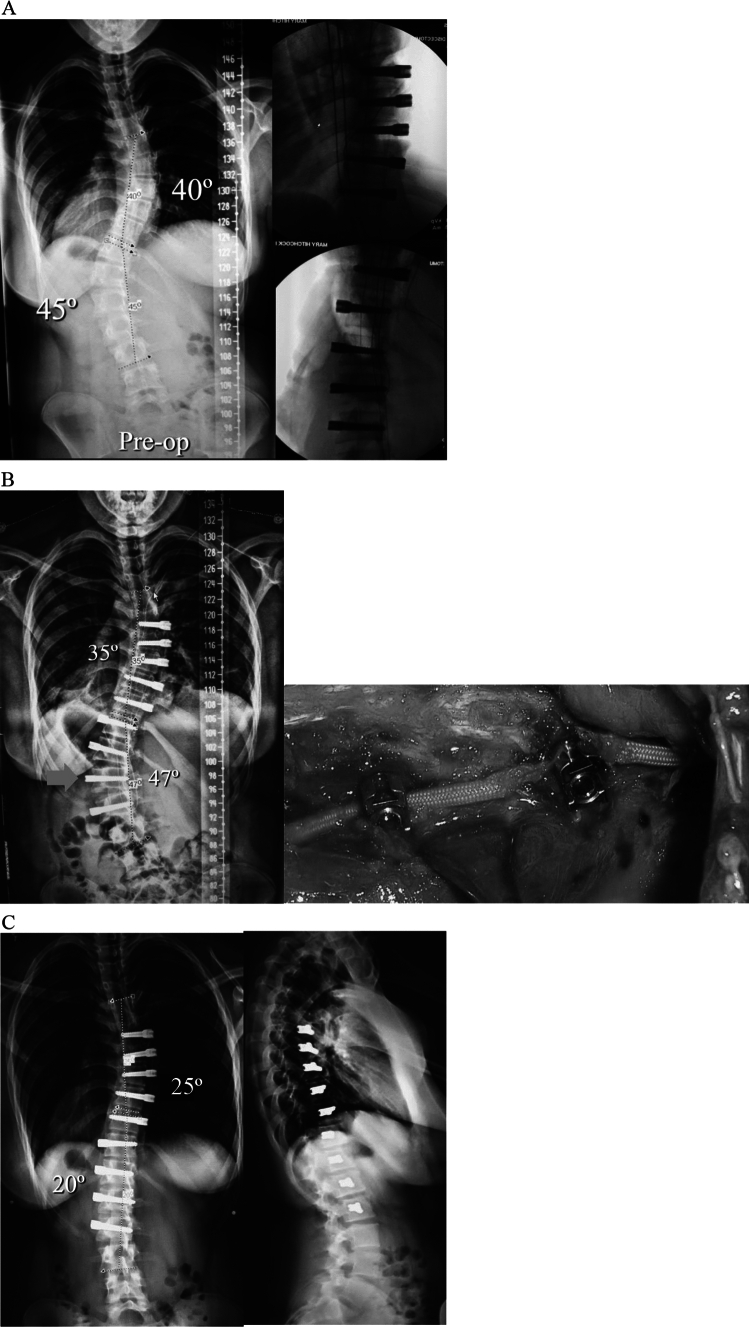
**A** Preoperative posteroanterior radiograph of a 14 + 11-year-old female with a Lenke 6C curve pattern involving a 40º curve right T6-T11 and 45º curve left T11-L3 at Risser 4 and Sanders 6 with significant correction of both curves to < 20º demonstrated on intra-operative fluoroscopy. **B** Postoperative posteroanterior radiograph after an audible pop was heard while ambulating on POD #2 demonstrating loss of correction of both curves but most significantly spanning T12-L2. An intra-operative photograph taken during revision surgery demonstrated tether rupture at the L1 screw. Analysis of the explanted Dynesys PET cord confirmed focal damage at the L1 screw site most likely related to overtightening of the Dynesys set screw with a non-torque-limiting screwdriver. **C** Postoperative posteroanterior and lateral radiographs demonstrating improvement of both curves to 25º and 20º, respectively, after immediate revision of the ruptured thoracolumbar tether only. Curve corrections were maintained at 2 year follow-up without further tether rupture issues

Multiple factors not analyzed in this study that could, indeed, contribute to tether rupture and, potentially, even revision surgery after tether rupture, include both implantation and patient-related factors that either decrease the integrity of the PET cord during surgery or increase stress on the PET cord after surgery. Implantation factors include: 1. PET cord damage during implantation (e.g., via overtightening of a set screw (Fig. 11) or multiple episodes of tightening and loosening of set screws on the PET cord during tensioning maneuvers; manipulation of the implanted portion of the PET cord with a vice grip or other crushing instrument; or implantation of the “non-implantable” portion of the PET cord); 2. PET cord damage after implantation (e.g., via abrasion from a bulging disc or impingement from a prominent endplate or rib); 3. High PET cord stress (e.g., related to inharmonious alignment of vertebral screws resulting in PET cord zig-zag in the sagittal plane; or related to a supra-stiff overall tether construct employing a vertebral screw-staple combination at all levels). Patient-related factors include: 1. obesity; 2. large, muscular athletes (especially collision athletes); 3. excessive immaturity or maturity.

While our overall tether rupture rate of 35% is somewhat less than the rates reported rates in most other studies [1–6], a simple explanation for this may not be possible. Though our patient population differs significantly from most other studies, in that it includes a broad range of curve magnitudes, curve types, and a full spectrum of skeletal immaturity to maturity, most of the factors that distinguish our study from others might actually create a greater risk of tether rupture rather than a reduced risk. One factor that we have considered in analyzing our decreased tether rupture rate is the potential benefit of using a vertebral screw alone for fixation at each level of a tether construct without staple augmentation. This practice differs significantly from almost all other centers performing tether surgery and might have some implication in the variable reported tether rupture rates. Though theoretical, we believe the use of a vertebral screw alone for fixation at each level of a tether construct, given appropriate bone quality, potentially reduces its overall stiffness, when compared to a vertebral screw–staple construct, and may protect the tether itself from high peak forces during physiologic motion.

Our rate of additional surgery after tether rupture was also found to be somewhat different in this study compared to other published reports. The rate of revision surgery in the 45 patients with tether rupture was 16% (7/45) with 9% (4/45) requiring tether revision alone and 7% (3/45) requiring conversion to fusion. The overall rate of revision surgery for tether rupture in all 262 patients in this study was 3% (4/133) for early tether ruptures and 2% (3/129) for late tether ruptures for a combined rate of 5%. Multiple other studies have reported a rate of additional surgery after tether rupture in the 30% range or higher with tether revision alone in up to 21% but conversion to fusion required in up to 24% of patients [1, 2, 10]. While the reasons for an increased rate of revision surgery and conversion to fusion are also difficult to define, the lower rate of additional surgery in our study may be related to our treatment of significantly more mature patients who likely had less risk for curve progression after tether rupture. Interestingly, the patients in this study with a single curve in the thoracolumbar/lumbar spine demonstrated the highest rate of tether rupture (21%) but had the lowest rate of revision surgery (0%), perhaps related to greater overall maturity in this group.

A comment on general complications and additional procedures after AVT seems warranted here to provide some context for the specific complication of tether rupture. Though multiple studies, including our own, have reported tether rupture as the most common complication after AVT, we found in our initial 10 year experience with this novel treatment that it was the least likely complication to require revision surgery or conversion to fusion [7]. The tether rupture rate in our first 52 patients treated with AVT over 10 years was 25%, but only 6% of these patients required revision surgery for tether rupture and none required conversion to fusion. In contrast, the complications of overcorrection and inadequate correction over our first 10 years were less common at 6% and 8%, respectively, but the majority of patients with these two complications required revision surgery with most requiring conversion to fusion. Fortunately, an evolution in our AVT indications and techniques over 14 years has resulted in a substantial reduction in these two other complications over time [11, 12]. And though our rate of tether rupture did not decrease in this current study, the requirement for revision surgery remained quite low at 5% with the majority of patients avoiding conversion to fusion.

An additional comment on complication profiles after AVT versus fusion surgery for AIS also seems appropriate given that AVT represents a novel alternative to the more standard treatment option of fusion. Though an initial analysis of these two procedures reveals a higher rate of complications and revision procedures after AVT versus fusion surgery, the complication rates for both are variable and overlap somewhat. For example, a number of AVT studies have reported complication rates as high as 50%, with revision surgery rates in the 25% range, while other AVT studies have reported complication rates as low as 12% with revision surgery rates in the same 12% range [1, 2, 10]. The reported rates of complications and additional procedures after posterior spinal fusion are lower, yet complications rates are still reported in the 5–23% range with revision surgery rates in the 6–12% range [13–15].

A more careful analysis of these two procedures, and the studies used to establish the risk profiles for each, suggests significant differences in the types of complications encountered and, perhaps, even their severity. Further, significant differences are evident in the maturity of the literature available to evaluate each procedure and in the overall priorities of these two treatments. First, though the risks of bleeding requiring transfusion, infection, significant neurologic injury, pseudoarthrosis, junctional kyphosis, adjacent segment degeneration, and even death seem low after posterior spinal fusion, these complications have not been reported after AVT. AVT simply does not carry the same risks as posterior spinal fusion. In our 14 year experience with this novel treatment, as well as in the experience of other major tether centers, the complications listed above have been avoided. And though the unique set of risks that accompany AVT—overcorrection, inadequate correction, and progression after tether rupture—can be problematic, they may, indeed, be less severe than some of the major complications associated with posterior spinal fusion. Second, there is an inherent bias in any comparison of an established procedure, like fusion, that has been practiced and honed for decades, to a new procedure, like AVT, that lacks decades of evolution and refinement. The supporting literature for posterior spinal fusion reflects not only an optimal level of surgeon experience but also maximally refined indications, techniques, and instrumentation systems. In contrast, the handful of AVT studies published to date essentially represent learning curve studies that lack the benefit of extensive individual or collective surgeon experience. These early AVT studies betray significant limitations in our general understanding of the ideal indications and techniques for this new procedure and rely on first-generation instrumentation systems that were repurposed from an adult lumbar spine instrumentation system. Though some authors have made an attempt to directly compare the earliest AVT results to selected posterior spinal fusion studies, these analyses, especially in the form of systematic review, are premature in their assessment of AVT and seem weighted in favor of fusion [16, 17]. Third, while AVT and fusion surgery share the same goal of significant deformity correction and control with the least negative impact on the normal physiology and biomechanics of the spine, the priorities for these two treatments differ significantly. Fusion prioritizes an instantaneous and permanent maximal correction of deformity using rigid metal rods and bone graft for fusion. AVT prioritizes a more flexible and physiologic correction of spinal deformity that seeks to preserve growth, motion, and function of the spine in the short term and reduce the risk of adjacent segment degeneration in the long term. More specifically, AVT prioritizes the avoidance of fusion. And while posterior spinal fusion shares the similar priority of minimizing the extent of fusion—by limiting fusion levels, saving lumbar levels, or selective thoracic fusion—these efforts to reduce the negative impacts of fusion do not provide the same reduction in fusion risk as the elimination of fusion altogether.

The limitations of this study are not insignificant. First, this is a retrospective study in which many patients lack 2 year follow-up data and a significant number of patients with 2 year data lack extensive follow-up into the 5–10 year range. It is likely that, with greater follow-up in a larger cohort of patients, the tether rupture rate would actually be higher than found in this study. Whether an increased tether rupture rate would result in a substantial increase in revision surgery remains to be seen. However, because this study merely represents a snapshot in time, it is important to exercise caution in interpreting these data. Though these data demonstrated a high combined rate of inconsequential and beneficial tether ruptures (71%) over the 14 year study period, these patients still remain at risk for additional consequential or problematic tether ruptures in the future. Second, the study lacked a patient-reported outcome instrument which might have provided greater insight into the convex back pain issues significantly associated with revision surgery. Despite these limitations, this study provides valuable insight into the most common complication after anterior vertebral tethering. A complication that often does not behave like a complication. While tether rupture is associated with a myriad of outcomes—good, bad, and ugly—most of these were found to be inconsequential or, occasionally, beneficial. Some, however, like consequential and problematic tether ruptures, remain concerning as they can require revision surgery and even result in conversion to fusion.
